# Using a Semi-Structured Qualitative Interview to Evaluate Pediatric Interns’ Use of the Electronic Medical Record in a Simulated Setting

**DOI:** 10.7759/cureus.14924

**Published:** 2021-05-09

**Authors:** Stefan Malin, Nathan Swinger, Emily Meanwell, Amelia Hawbaker, Kamal Abulebda

**Affiliations:** 1 Pediatric Critical Care Medicine, Riley Hospital for Children at Indiana University Health, Indianapolis, USA; 2 Sociology, Indiana University, Bloomington, USA

**Keywords:** electronic medical record, emr, simulation, training, medical education

## Abstract

Introduction

Effective use of electronic medical record (EMR) is paramount to delivering safe and effective care. Current EMR education is inadequate, with literature showing frequent deficiencies in skills needed to obtain and interpret data. This study aims to evaluate pediatric interns' perception of EMR inclusion in scenario-based simulation training.

Methods

A total of 13 pediatric interns participated in an EMR-enhanced, multidisciplinary simulation of a pediatric patient with septic shock during the 2019-2020 academic year. Following the simulation, the interns participated in a semi-structured interview to evaluate the experience of having the EMR incorporated into the simulation and what benefits it offers.

Results

Of the 13 interns, 12 (92%) felt that incorporating the EMR into the simulation increased the realism of the scenario. All (100%) interns reported that EMR inclusion led to increased learning about the EMR, including gaining or re-learning skills needed to access or interpret electronic clinical data. Participants felt that EMR inclusion in the simulation provided valuable learning opportunities not present in traditional EMR education.

Conclusions

Integrating the EMR into simulation is viewed positively by pediatric interns, is perceived to improve simulation realism, and helps teach important EMR skills. EMR training would benefit from incorporation into scenario-based simulations.

## Introduction

The electronic medical record (EMR) is one of the primary sources of information in many clinical settings. Its utilization among clinicians has improved adherence to guideline-based care, disease surveillance and monitoring, and decreased medication errors [1,2]. However, there can be challenges using a nonintuitive platform and filtering a large amount of data to discern what is clinically relevant, leading to potential patient harm [3-6]. Magnifying challenges of EMR use include the relative lack of provider training [7-9]. Providers often receive less than three days of EMR training, yet research shows that more extensive training is often needed [8,9]. Further, EMR training often relies on passive learning methods, which often do not lead to a successful transfer of skills to the clinical environment [10,11].

Simulation has been used for some forms of EMR training. Studies have used simulation to train utilization of specific EMR applications, such as a data visualization tool, and as a means to evaluate EMR user proficiency [11-13]. In some of these studies, trainees failed to identify serious medical management issues and concerning vital sign trends [11,13]. These findings highlight a potential gap following standardized EMR training. Little is known about medical trainees’ perception of the value of EMR in patient care, its role in clinical decision-making, and the barriers to its practical use. While simulation-based EMR curricula could likely add significant value, fully understanding learners’ perceptions of the traditional curriculum and the added value of high-fidelity simulations would better target these interventions. In this study, we aim to explore early-career pediatric interns’ perceptions of EMR inclusion in simulation and its role in EMR training in a simulated setting. We hypothesized that EMR inclusion would improve the realism of the simulation and enhance EMR data acquisition and interpretation skills.

## Materials and methods

Study design

This is a prospective qualitative study to evaluate pediatric interns' perception of EMR integration with simulation. This study was deemed exempt by the Indiana University Institutional Review Board.

Study population and settings

All pediatric interns, including those from combined programs, on their complex care inpatient rotation were recruited over a five-month period (August 2019 to January 2020). The simulations involved one intern at a time. Inpatient ward nurses were also recruited to participate as a bedside nurse during the simulation sessions.

All simulations were carried out in a patient care room on the inpatient ward of a large metropolitan hospital. Each simulation session began with a standard orientation to the study and simulator, SimJunior® (Laerdal Medical, Wappingers Falls, NY). The participants were also introduced to the confederate ‘‘parent’’ (played by one of the authors). The study team utilized scripted information and answers to common questions. The scenario consisted of a pediatric patient with sepsis and compensated shock. This scenario was chosen so that interns needed additional information in the EMR to make a diagnosis. Participants were provided with a brief medical history: a four-year-old male with a history of cerebral palsy, developmental delay (including being nonverbal), feeding dysfunction, and gastrostomy tube dependence who was admitted 48 hours before the scenario for feeding intolerance, vomiting, mild dehydration, and a viral syndrome. Before the beginning of the simulation, the resident and nurse received a structured sign-out stating that the patient was in stable condition, tolerating Pedialyte® with minimal vomiting, and was likely to be discharged the following day. The intern and nurse were also instructed that they had access to the patient's chart in the EMR and could use the workstation on wheels in the room. The simulation began with the nurse recording the vitals of the patient for the proceeding hour. For the sake of this scenario, the intern was tasked with the initial management of the patient without immediate availability of a more senior physician. Each simulation lasted approximately 15 minutes.

During the simulation, the intern and the nurse had access to a workstation on wheels with the simulated patient’s chart in the EMR (Cerner©). The simulated chart was created by the study team. The chart included the patient’s vital signs, urine output, and fluid balance over the preceding 48 hours. These revealed worsening tachycardia, low normal systolic and diastolic blood pressures, and oliguria. The intern and nurse were also instructed to use the EMR exactly as they would in a real patient encounter. Following the simulation, the principal investigator conducted a facilitated debriefing that focused on participants’ utilization of EMR within the scenario and how the EMR can aid in diagnosing and managing a child with sepsis.

Qualitative interviews

Each intern participated in a qualitative, semi-structured interview to assess their overall experiences with the EMR in the simulation, including new information learned or the reinforcement of previous training that may inform future practice. They were also asked to reflect on previous EMR training, including areas of self-identified proficiency or weakness. Audio recordings of the interviews were transcribed using a professional transcription service and averaged 10:48 minutes in length. An independent sociologist with expertise in qualitative research, conducted a thematic analysis of the transcript. A multi-stage approach was used to code and analyze the interview data, beginning with an initial phase of open coding [14,15]. Structural, or index, codes based on the interview guide were also applied to the transcripts. Based on this initial coding, the analyst identified and categorized prominent themes and created a codebook with definitions of each theme. Finally, based on this coding, the analyst generated several analytic memos related to the contribution of EMR inclusion to simulation realism and medical learning. These memos were then reviewed by a second qualitative researcher and the primary research team for accuracy.

## Results

Adding EMR to simulation improves realism

Of the 13 interns, 12 (92%) explicitly described the simulation as realistic (Table 1). Utilizing EMR during the simulation was described by interns as contributing to a sense of realism in several different ways, including the simulation feeling more like a true patient encounter, having to obtain information from the EMR, and synthesizing this data in relation to the patient.

**Table 1:** Realism of the Simulation Summary of themes by interview respondent for codes included with “EMR and realism.” Dots indicate whether or not each intern discussed the theme during the qualitative interview. The total number and percentage of interns who discussed each theme are reflected at the bottom. EMR, electronic medical record

Intern	Discussion of Realism (General)	Realism: Themes					
		General/Nonspecific	EMR/Computer	Parent	Staffing	Sub-Acute	Other
1	•						
2	•	•					
3	•		•				
4	•		•				
5	•		•				
6	•	•					
7	•	•	•				
8	•		•				
9	•		•	•		•	•
10	•		•				•
11	•		•				•
12	•		•	•	•		•
13	•		•			•	•
Total	13	3	10	2	1	2	5
%	100%	23%	77%	15%	8%	15%	38%

In the interviews, interns described the inclusion of EMR in this simulation as contributing to a more realistic feeling than other simulations in which they had previously participated. They noted that in past simulations, key pieces of information, such as vital signs, were provided on pieces of paper or in response to the simulation participant asking the proctor for the information. For example, one respondent commented, “I think in most sims usually there's just a thing on the door that says what it is, and then you get a piece of paper that has the vital signs on it and things like that, so I think it was a more realistic set up in this situation than in my prior situations” (respondent 12). Another intern explained, “I haven't done a lot of simulations in the past, but typically you ask for what the vital signs are, and you get them, and so it's a much, this is still a pretty truncated version of what may happen, but I felt like it did add a much more realistic element, because then you're actively doing it and you're actually utilizing the record, and that's something that would happen in real life…” (respondent 7).

EMR-related time consumption was also discussed as contributing to a sense of realism. For example, one intern talked about the inclusion of the EMR as making the simulation more realistic in terms of time: “…the simulation I’ve done before, it hasn’t ever included the computer. It’s been I’m ordering this; you’re saying it out loud, and then whosever proctoring it gives it back to you versus it actually takes me a second to put it in the EMR” (respondent 5). The “truncated” version referenced above in most simulations was discussed in terms of time and the amount of information provided. One respondent explained in detail: “You actually are going through the process that you normally would in a patient room, and you have extraneous information in the EMR, whereas typically in all the other simulations that I've been in, you're just asking someone back and forth, so they'll just say oh, that's not relevant…they would just maybe tell me, oh yeah, their blood pressures have kind of been trending up, or this was his last one, a few hours ago, so it gives a much more full picture and a more realistic picture, without having the bias of someone giving you only the information that you need, and interpreting it a little bit for you” (respondent 10).

Incorporating the EMR into the simulation was not the only factor interns described as contributing to its realism. One respondent described this simulation as more realistic than others because it included a real nurse; two respondents also mentioned having a parent in the room (played by one of the facilitators) as an additional factor enhancing the simulation’s realism.

Learning about EMR through simulation

In addition to reporting that the EMR contributed to the realism of the simulation, participants also indicated that the simulation helped them learn to use the EMR. All interns reported learning about the EMR in some manner during the simulation (Table 2). Common topics within this theme included learning how to use the EMR to better understand patient trends, primarily through visualization tools; learning new skills in the EMR and being reminded of previously-learned but forgotten skills; and the importance of hands-on technical learning on how to navigate the EMR in comparison to other EMR learning opportunities.

**Table 2:** Learning about EMR through Simulation Summary of themes by interview respondent for codes included with “Learning about EMR via Simulation.” Dots indicate whether or not each intern discussed the theme during the qualitative interview. The total number and percentage of interns that discussed each theme are reflected at the bottom. EMR, electronic medical record

Intern	Learning about EMR via Sim (General)	Learning about EMR via Sim: Themes						Learning via Other Mechanisms
		Graphs/Visuals	Trends	Hands-On	New	Reminder, Refresher	Other	
1	•	•	•					
2	•				•			
3	•			•	•		•	
4	•	•	•	•	•	•		
5	•	•	•	•			•	•
6	•			•		•		•
7	•	•	•	•		•		
8	•		•			•		
9	•							
10	•	•	•	•	•	•		
11	•	•	•				•	
12	•						•	
13	•	•			•		•	•
Total	13	7	7	6	5	5	5	3
%	100%	54%	54%	46%	38%	38%	38%	23%

Seven (54%) interns reported how EMR learning helped them better understand patient trends and visualization tools. During the simulation, no intern was observed to trend 48-hours of vital signs, urine output, or total intake and output. Very few interns were even observed to evaluate the preceding six hours of vital signs. One intern stated, “It definitely would have been helpful to look at the trend of the vitals, the tachycardia going up and the blood pressure going down” (respondent 1). Another reported, “when we expanded the vital sign report to show the trend, you could see that he was out of what his baseline was” (respondent 4). Seven (54%) interns also referenced the importance of looking at trends, with three explicitly stating that they would utilize this feature of the EMR in the future. One intern reported that “it’s better than just looking at numbers, you can actually see if, for example, heart rate or blood pressure is down-trending, and by how much” (respondent 11).

Aside from graphing vital signs, three (23%) interns also discussed learning how to graph urine output in the EMR during the simulation. One intern recalled that “I really like knowing the urine output, that the graph is there…I didn’t realize that was available in Cerner” (respondent 3). Learning where this function was found in the EMR was a skill the intern learned during the debriefing. Another intern reported that the in-and-out flowsheet is “busy and confusing” and that “I’ve never seen that before, and it’s such a quick way of seeing it” (respondent 10).

Five (38%) interns commented that these were new skills that they learned through the simulation, while five (38%) interns commented that these were previously learned skills that had not been used consistently (three interns did not comment on whether these were new or old skills). One respondent noted, “I did like the thing that you showed us on how to view the trends, because I always forget about that” (respondent 8). When barriers to utilizing the skills gained during the simulation were explored, the theme of forgetting previously taught skills became more evident. One intern replied, “I think I need to see it in practice more regularly” (respondent 13), with another commenting on needing reminders and practice until it becomes routine. One intern ultimately concluded, “the more practice we get with simulations, I think it will just become second nature” (respondent 11).

Six (46%) interns discussed the hands-on quality of learning that took place during the simulation. One respondent found it helpful to incorporate the EMR, “especially in this setting, just to get more comfortable with how to use it” (respondent 7). One intern noted that it is important to learn so that you can “troubleshoot your problems that the EMR might be giving you” (respondent 3), while another described the simulation as a “test of my ability to use the EMR” (respondent 5).

Finally, respondents discussed EMR learning through simulation and compared it to previous models through which they have been taught the EMR. One intern noted that having an EMR refresher course might be more helpful: “these are the problems I encounter, so when they’re telling me and teaching me about them, I actually know what to ask for and can actually follow along with what they’re doing” (respondent 6). This idea of learning, not only through formal teaching but also through on-the-job training, was also expressed by others in less concrete terms, such as one intern commented that “I think I need to see it in practice more regularly” (respondent 13).

## Discussion

This study offers an important view into the potential benefits of EMR integration in simulation and the creation of an EMR-based simulation curriculum. This study identifies two key factors: EMR inclusion in a simulated learning environment improves the realism of the simulation and enhances EMR utilization through obtaining or re-learning EMR skills.

Interns reported that the EMR made the simulation more realistic. Incorporating the EMR into high-fidelity scenarios more closely replicates the clinical environment and creates a more optimal learning environment. Increasing the realism of the way information is presented also forces participants to locate the information and evaluate its relevance. Having to use the EMR during this simulation also replicated some of the cognitive load that interns are subjected to when encountering a real patient’s chart. This allowed our learners to understand and reflect on the function, challenges, and value of EMR utilization. This study supports the inclusion of the EMR into simulated learning as an enhancement to realism.

Secondly, EMR inclusion allowed participants to re-learn specific EMR skills or to obtain new skills. During this simulation, participants did not utilize information in the EMR that may have aided their diagnostic evaluation because they did not know or could not remember how to find it. Simulated patient care involving the EMR provides interns with practice integrating the EMR into a realistic patient encounter while allowing an opportunity to work through the nuances of the EMR. During the interviews, interns expressed the value of the EMR in evaluating patient vital signs and trends that helped them understand the progress of the clinical condition. This simulation allowed interns to learn or re-learn EMR skills and augmented any past learning that may have occurred. Future studies evaluating the persistence of learned skills and knowledge in both simulated and actual patient encounters will be needed.

This study was limited by the number of participants who were able to complete the simulated scenario. While all pediatric interns were eligible to participate, the emergence of the SARS-CoV-2 (severe acute respiratory syndrome coronavirus 2) pandemic truncated the study and we were unable to complete additional simulations after January 2020. Another limitation was our inability to create a simulated patient chart that was used exclusively for this study. In our health system, test patient charts can be used by anyone with EMR access. Consequently, during the simulations, extraneous information not related to the simulation was occasionally found by the interns. If this happened, interns were instructed to ignore it. No intern said that it impacted the simulation or their ability to care for the simulated patient; however, a dedicated simulated EMR patient would allow for an even more immersive experience and allow refinement of simulated cases.

## Conclusions

Qualitative data from this study demonstrate that incorporating the EMR into a simulated learning environment improves the simulation realism and helps enhance EMR skills among pediatric interns. EMR inclusion in simulation helps users learn new skills for EMR data acquisition and interpretation. Additionally, it can help strengthen previously learned skills. Given the limitations in current training methodologies for the EMR, these findings highlight the need for continued and improved training around EMR and support simulation as a mode of EMR training.

## Appendices

Prior Exposure to EMR

1.      Tell me about the last time you used EMR in resuscitation or management of ill patients. (If never been used like this before, then skip to the current simulation encounter and change wording and questions appropriately).

a.      What went well?

b.      What were some of the facilitating factors that helped it go well?

c.      What could have gone better?

d.      What were the barriers you faced when caring for the patient?

e.      Did you feel there was information that you wish you knew but you didn’t?

Current Simulation

1.      How many years of experience do you have utilizing Cerner?

2.      Did you utilize information in the EMR to aid in diagnosis/management?

a.      What information did you use?

b.      How did this information help you?

c.      If you didn’t use the EMR, how could you have utilized it and how would it have helped you?

3.      How do you feel your prior experience with EMR helped you out in the simulation session you participated in today? (if relevant)

4.      How was this case different than your previous EMR patient?

5.      Were you able to identify any gaps in knowledge that the EMR would have helped with?

6.      On a scale of 1-10, how well do you think you were able to identify the priorities in care for this patient?

a.      Why did you not rank yourself higher?

b.      Why did you not rank yourself lower?

7.      Have you participate in simulation in the past?

a.      What kinds of simulation?

b.      Have you ever participated in a simulation for a patient with sepsis or shock?

c.      Have you ever participated in a simulation that utilized the EMR?

8.      Do you think incorporating the EMR into this simulation added anything? 

a.      What did it add?

b.      Did it enhance the realism of the simulation?

Future Use

1.      Do you think you will be able to take the information gained today and implement it in future cases?

a.      Why or why not?

b.      What needs to happen in order for this to occur?

c.      What facilitators are present?

d.      What are the barriers?
